# The Role of Epigenetics in Arterial Calcification

**DOI:** 10.1155/2015/320849

**Published:** 2015-06-29

**Authors:** Shan-Shan Wu, Xiao Lin, Ling-Qing Yuan, Er-Yuan Liao

**Affiliations:** ^1^Endocrinology Department, Pingxiang People's Hospital, 128 Anyuan Area Square Road, Pingxiang, Jiangxi 337000, China; ^2^Institute of Metabolism and Endocrinology, The Second Xiangya Hospital, Central South University, 139 Middle Renmin Road, Changsha, Hunan 410011, China

## Abstract

Arterial calcification is highly prevalent and correlated with cardiovascular mortality, especially in patients with ESRD or diabetes. The pathogenesis of arterial calcification is multifactorial, with both genetic and environmental factors being implicated. In recent years, several mechanisms contributing to arterial calcification have been proposed. However, these can only explain a small proportion of the variability in arterial calcification, which is a major obstacle for its prevention and management. Epigenetics has emerged as one of the most promising areas that may fill in some of the gaps in our current knowledge of the interaction between the environmental insults with gene regulation in the development of diseases. Epigenetics refers to heritable and acquired changes in gene transcription that occur independently of the DNA sequence. Well-known components of epigenetic regulation include DNA methylation, histone modifications, and microRNAs. Epigenetics research in the regulation of arterial calcification has only recently been elucidated. In this review, we will summarise recent progress in epigenetic pathways involved in arterial calcification and discuss potential therapeutic interventions based on epigenetic mechanisms.

## 1. Introduction

Arterial calcification (AC), a crucial pathologic component of vascular diseases such as atherosclerosis, coronary artery disease, and peripheral vascular disease, is far more common among patients with end-stage renal disease (ESRD) and diabetes compared with the general population [1–4]. AC not only impairs vasomotor responses, but also influences the stability of atherosclerotic plaques which are prone to rupture, particularly in regions of high background stress, with microcalcifications located in the thin fibrous cap [5–7], eventually leading to myocardial infarction. Thus, AC is a potentially life-threatening condition and understanding the causes of arterial calcification may contribute to the treatment and possibly prevention of this disease. However, there is no available therapy that could reverse arterial calcification at present, even with the recent therapeutic progression, such as bisphosphonates. At present, available therapy just can slow down the progress of arterial calcification. Thus, revealing the pathophysiological mechanism of AC and finding novel therapies that reverse the progress of the vascular remodelling are our target in treating this disease.

AC has been recognised for over a century. Unravelling the mechanism involved has been a topic for many researchers in the past few years. Previously, AC was regarded as a passive, degenerative, end-stage process accompanied by calcium-phosphate mineral precipitation in vessel walls. However, increasing evidence has shown that AC is an active and tightly regulated event that is analogous to mineralisation in bone tissue [8–11]. This is based on the discovery of phenotypic conversion of vascular smooth muscle cells (VSMCs) into osteoblast-like cells, as evidenced by the expression of bone-regulating proteins such as alkaline phosphatase, osteocalcin, and Runx2 (runt-related transcription factor 2)/Cbfa1. Several excellent reviews have been published on AC [12–14], manifesting the vital roles of molecular and genetic factors in this complex disorder. Currently, we are fully aware of the important involvement of epigenetic processes in the regulation of gene expression. Understanding these processes is critical for further insight into the pathogenesis and development of AC.

Since Conrad Waddington first coined the term “epigenetics” back in 1942, research has advanced from genotype to phenotype [15]. Epigenetics refers to heritable alterations in gene expression without alterations in the genetic code itself [16]; such alterations regulate the dynamics of gene expression and play a crucial role in embryonic development, imprinting, and tissue differentiation [17]. Recent breakthroughs in the field of epigenetics offer us a new perspective on gene regulation, which broaden the conventional cis/trans paradigm of transcriptional processes and transform our conceptualisation of the impact of the environment upon our genes and health [16, 18]. Epigenetics regulation is composed of three main categories: DNA methylation, histone modification/chromatin remodelling, and microRNAs (miRNAs) [19]. However, the group is likely to be expanded in the future [20]. Numerous lines of evidence have implied that epigenetic processes play crucial roles in the development of various diseases (cancers, neurological disorders, autoimmune diseases, and diabetes) [21, 22]. Research investigating the actual role of epigenetics in arterial disorder remains scarce; however, several emerging lines of evidence suggest that epigenetics may be important in the biology of VSMCs and the pathogenesis of arterial calcification. This review will summarise the current knowledge about these subjects.

## 2. Arterial Calcification

### 2.1. Mechanisms of Arterial Calcification

The mechanism of AC is complicated. It is not simply composed of precipitation of calcium (Ca) and phosphate (P) but rather is an active and modifiable process in which the VSMCs undergo changes from contractile to secretory phenotype, inducing matrix formation and also attracting local factors that are involved in the mineralisation process. Several different mechanisms for initiating AC have been proposed. First, human and mouse genetic studies have found that blood vessels normally express inhibitors of calcification, indicating that AC is generally inhibited by the physiological function of resident arterial cells. Deficient expression of even one inhibitor of AC is enough to trigger the calcification process [23, 24]. Calcification inhibitors such as matrix GLA protein (MGP) may restrain AC by binding to bone morphogenic proteins (BMP-2) [25]. Likewise, fetuin-A is the most potent circulating inhibitor of extraosseous calcification. Decreased fetuin levels have recently been associated with elevated CVD mortality in haemodialysis patients [26]. Apart from GLA protein or fetuin-A, various other factors have been related to arterial calcification. Among those, BMP-7, osteoprotegerin, osteopontin, and inorganic pyrophosphate, an inhibitor of hydroxyapatite crystal growth, probably counteract detrimental vascular and soft-tissue calcification in CKD [27–29], while BMP-2, RANKL, and leptin have been found to promote arterial calcification [30–32]. Second, the death or “damage” of VSMCs can provide phospholipid-rich membranous debris and apoptotic bodies that may serve as initiation sites for apatite crystallisation [33], particularly in diseases where necrosis and apoptosis are prevalent, such as atherosclerosis [34, 35]. Third, we and others have provided evidence that AC is a process reflecting the transformation of the VSMCs from contractile to secretory phenotype [11, 36–38]. In this process, osteoblast-specific genetic programs are triggered with the presence of osteopontin, BMPs, osteocalcin, and Runx2/Cbfa1 [39], which result in the formation of mineralised matrix, cartilage, and bone, and suggest that osteogenic mechanisms may also play an important role in arterial calcification. The phenotypic modulation of VSMCs can be induced by increased extracellular Ca and P content [40, 41] as well as by various other modulators, such as proinflammatory cytokines (e.g., IL-6, TNF-a) [42], oxidised lipids [43], and microenvironmental and mechanical cues [44]. Finally, accumulating serums Ca, P, and Ca × P are associated with arterial calcification and cardiovascular mortality in patients with ESRD via thermodynamic mechanisms. Elevating Ca or P levels in the culture media leads to enhanced mineralisation and phenotypic changes of VSMCs characterised by a decrease of smooth muscle-specific gene expression and the upregulation of genes associated with bone differentiation [40, 41]. Elevated calcium-induced mineralisation and P-induced phenotypic transition and mineralisation were found to be dependent on the function of a sodium-dependent phosphate cotransporter, Pit-1, based on their ability of being inhibited by phosphonoformic acid [41] and Pit-1-specific small interfering RNA [45]. Briefly, a deficiency of calcification inhibitors, cell death, phenotypic transformation of VSMCs to osteoblastic cells, or/and disturbance in Ca and P metabolism may all initiate and sustain arterial calcification in a concerted manner.

### 2.2. Pathology and Clinical Consequences of Arterial Calcification

AC is a pathological process that occurs in response to dysregulated or inappropriate environmental stimuli such as advancing age, atherosclerosis, and some metabolic disorders (e.g., CKD, diabetes, and chronic inflammatory disease) and in rare genetic diseases (e.g., Keutel syndrome) [31, 42]. AC develops at two anatomic sites: intima and media layers of the large and medium-sized arterial wall [46]. Intimal calcification occurs in atherosclerotic plaques and progresses in parallel with the plaque evolution [47, 48]. In contrast, medial calcification (also known as Monckeberg's medial sclerosis) could take place independently from atherosclerotic plaques and has been observed in the context of aging, diabetes, and ESRD [49–51]. However, both types of arterial calcification often develop simultaneously in dysmetabolic patients. Intimal calcification is characterised by lipid deposition and macrophage accumulation and has been associated with inflammatory cells and VSMCs. In contrast, in medial calcification, metabolite-induced arterial changes in the absence of lipid deposits or macrophages are viewed as specifically accounting for the upregulation of osteogenic regulatory genes which then induce osteogenic differentiation of mesenchymal cells with following matrix mineralisation, bone, and cartilage formation [42]. Media calcification causes arterial stiffness, linked with high pulse pressure (characterised by increased systolic and decreased diastolic pressure), left ventricular hypertrophy, and reduced coronary perfusion [52]. While intimal calcification mainly results in occlusion of vessels, nevertheless, the consequences of intimal calcification on plaque vulnerability remain less clear, as the determinants of plaque rupture with consecutive thrombosis are still controversial. Several researches have implied that AC does not elevate plaque vulnerability, which seems more ascribable to a large lipid pool, thin fibrous cap, and intensity of local inflammation [7, 53]. In the end, both count, and they are partly responsible for the morbidity of atherosclerosis, acute coronary events, and even heart failure.

### 2.3. Epigenetics and Its Roles in Arterial Calcification

Epigenetics was first proposed by Conrad Waddington in a study of “the causal interactions between genes and their products, which bring the phenotype into being” [54]. More recently, epigenetics was redefined as the study of stable changes in gene expression without alterations in the DNA sequence [16]. Such changes are achieved by covalent and noncovalent modifications, which mark the genome and play a role in turning genes on or off [55]. The most well-known epigenetic mechanisms include DNA methylation, histone modification/chromatin remodelling, and miRNAs.

The vascular system is highly regulated by epigenetic mechanisms. Growing evidence has shown that epigenetic markers exert a crucial role in vascular development, endothelial and smooth muscle cell differentiation and function, and allowing a high flexibility in response to sudden physiological or pathological changes. Epigenetic factors also explain how external factors such as diet, environment, and lifestyle may contribute to cardiovascular disease. However, reports on the roles of epigenetics in AC have only emerged in recent years.

### 2.4. DNA Methylation

DNA methylation, the first mechanism involved in epigenetics, is the most widely studied epigenetic marker [56]. In mammals, DNA methylation occurs mostly within CpG dinucleotides via the addition of a methyl group from SAM to the fifth carbon of a cytosine residue to form 5-methyl-cytosine. The CpG dinucleotides are inclined to cluster in regions called CpG islands, which are identified as a region of more than 200 bases with a CG content of at least 50% [21]. CpG dinucleotides are usually underrepresented in mammalian genomes (~1%). Approximately 70% of human gene promoters reside within CpG islands and are generally unmethylated in normal cells [57]. Some tissue-specific DNA methylation, however, takes place in regions termed CpG island shores as far as 2 kb distant of the promoter region. Furthermore, cancer-specific methylation also occurs at conserved tissue-specific CpG island shores [58]. The basic mechanism of DNA methylation is summarised as follows: enzymes catalyse the addition of methyl groups onto cytosine residues, enzymes modify and remove the methyl group, and then proteins recognise and bind to methyl groups to ultimately influence gene expression. So far, three active DNA methyltransferases (DNMTs), which directly catalyse the addition of methyl groups onto DNA, have been described: DNMT1, DNMT3a, and DNMT3b [21]. DNMT3L, which belongs to the DNMT3 family, lacks the catalytic activity itself, but it is required for the enzymatic activities of DNMT3a and DNMT3b and interacts with them in the nucleus [59, 60]. DNMT2 is a highly conserved protein and possesses tRNA methyltransferase activity [61]. DNMTs are capable of both methylation and demethylation, making the modification reversible [62, 63]. DNA methylation-related proteins, including the MBD (methyl CpG-binding domain) proteins, the UHRF (ubiquitin-like, containing PHD and RING finger domain) proteins, and zinc-finger domain proteins, can bind to 5-mC with a high affinity to inhibit transcription factor binding [64]. DNA methylation represents a hallmark of gene silencing. The mechanism of silencing can be mainly through (1) the promotion of methylated DNA in the recruitment of methyl-CpG-binding domain (MBD) proteins which, in turn, recruit histone-modifying and chromatin-remodelling complexes to methylated sites [65, 66] or (2) a preclusion in the recruitment of DNA binding proteins from their target sites [67].

### 2.5. DNA Methylation in Arterial Calcification

High phosphate concentration is tightly related to AC in patients with chronic kidney disease. de Oca et al. found that DNMT activity and methylation of the promoter region of the smooth muscle cell-specific protein SM22a increased with high phosphate concentration (3.3 mmol/L), using two* in vitro* models [68]. This was accompanied by a loss of SM22a, a gain of the osteoblast transcription factor Cbfa1, and the increased activity of ALP with subsequent* in vitro* calcification. They also demonstrated that procaine (a demethylating agent) decreased DNMT activity and methylation of the SM22a promoter, which was accompanied by upregulation of SM22a expression and less calcification. In addition, downregulation of SM22a by siRNA or a methyl group donor (S-adenosyl methionine) led to the overexpression of Cbfa1 [68]. This study was the first to link DNA methylation to the loss of smooth muscle lineage marker SM22a in VSMCs incubated with high phosphate and provided epigenetic mechanisms underlying phosphate-induced calcification of VSMCs.

### 2.6. Histone Modification and Chromatin Remodelling

The next breakthrough came with the identification of histone modifications in the mid-1990s, after which the DNA world developed from one dimension (linear sequence of base pairs) to three dimensions (nuclear topology), with the realisation of the important role of chromatin structure in regulating the genome [69]. The basic unit of chromatin, the nucleosome, is comprised of an octamer of four core histone proteins (H2A, H2B, H3, and H4) which are wrapped around a 147 bp segment of DNA in 1.65 left-handed turns [55]. Histone proteins within the nucleosomal core are predominantly globular except for their N-terminal “tails,” which are unstructured and subject to modification [55]. At least eight distinct types of modifications occur on histone tails: acetylation, methylation, phosphorylation, ubiquitination, SUMOylation, ADPribosylation, deimination, and isomerisation. Histone modifications play vital roles in transcriptional regulation, for instance, chromosome condensation [55], DNA replication, alternative splicing [70], and DNA repair [71]. In contrast to methylation, histone modifications are more dynamic and are indirectly correlated to gene silencing or activation [72]. Furthermore, histone modifications are tightly associated with the context in which they occur and the presence of additional modifications, indicating the existence of a “histone code” [72, 73]. Among the modifications, histone acetylation is the most well-studied. Histone acetylation takes place on lysine (K) residues and refers to the transfer of an acetyl group from acetyl-coenzyme A complexes. In mammals, this reaction is exerted mainly by three histone acetyltransferase (HAT) families, including CBP/p300, GNAT, and MYST [55, 74]. This modification often occurs along with transcriptional activation via stabilising the basic charge of lysine residues and reducing their affinity for DNA and finally preventing the formation of highly condensed chromatin in some cases [74–76]. In contrast, the effect of HAT could be antagonised by the opposing histone deacetylase (HDAC) enzymatic activity which mediates the removal of acetyl groups from lysine residues. At least four classes of HDACs have been identified: class I (HDAC1–3, HDAC8), class II (HDAC4–7, HDAC 9-10), class III sirtuins (SIRT1–7), and class IV (HDAC11) [74]. These HDACs are Zn^2+^-dependent, except class III HDACs, which are NAD-dependent. This enzymatic activity has been related to cell-cycle progression, gene silencing, differentiation, and DNA damage-induced response [76]. Since HATs and HDACs are two large enzymes families of antagonistic actions, the balance between them accounts for a pivotal regulatory mechanism for gene expression, developmental processes, and disease progression. In addition to histone modifications, chromatin can be remodelled by ATP-dependent chromatin remodelling complexes [77], which make use of ATP hydrolysis to alter the histone-DNA interaction so that nucleosomal DNA becomes much more accessible to interacting proteins [78]. These variations in chromatin structure result in changes in transcription in numerous sorts of biological processes and provide enormous modifications of functional responsiveness.

### 2.7. Histone Modification in Arterial Calcification

The switch of VSMCs from a contractile to synthetic phenotype is controlled by a series of transcription factors, particularly serum response factor (SRF) and its main cofactor, myocardin [79]. Almost all SMC-specific protein genes and genes that are important for SMC phenotypic switching contain the CArG box DNA sequence within their promoter [80]. Binding of SRF to the CArG box sequences activates VSMCs-specific contractile genes. Recent data indicate that the SRF binding in SMCs is modulated at the level of chromatin structure. Experiments* in vivo* and* in vitro* have shown that the binding of SRF to CArG boxes of SMC-specific genes such as SM22 is highly dependent on histone modifications [81, 82]. These include acetylation of lysine 9 in histone H3, acetylation of histone H4, and dimethylation of lysine residues 4 and 79 in histone H3. Moreover, deacetylation of histone H4 was accompanied by a loss of SRF binding to CArG box DNA during attenuation of SMC differentiation in response to vascular injury [82]. SRF-mediated transcriptional activation could be enhanced by its coactivator myocardin via interaction with the MADS box of SRF. Myocardin can recruit p300, a HAT that is associated with the transcriptional activation domain of myocardin and increases the transcriptional activity of specific genes [83]. In contrast, class II HDACs suppress smooth muscle gene activation by interacting with a domain of myocardin different from the p300-binding domain.

### 2.8. miRNAs

A newly discovered epigenetic mark involves miRNAs, a novel class of small, noncoding RNAs that are not translated into proteins [84]. miRNAs are initially transcribed in the nucleus by RNA polymerase II or III, forming long precursor transcripts [85]. These precursor RNA molecules subsequently undergo a specific cleavage driven by the Drosha and Dicer enzymes in the nucleus and cytoplasm, respectively. The resulting mature miRNA is single-stranded and composed of 18–22 nucleotides. miRNA is loaded into a miRNA-induced silencing complex (RISC) and directed to the target mRNA by pairing with sequences in the 3′UTR of target mRNA, leading to either degradation of mRNA or translational suppression. Studying miRNAs and their effects on translation is one of the most novel and active areas of epigenetic research. Approximately 1500 different miRNAs have been identified in humans so far, and the number will probably increase in the future [86]. The potential impact of miRNA-mediated biological regulation is estimated to be considerable. Numerous miRNAs have been found to be critical modulators of vascular pathologies, such as atherosclerosis, arterial remodelling, angiogenesis, and apoptosis [87].

### 2.9. miRNAs in Arterial Calcification

The research into miRNAs is rapidly growing and recent studies have disclosed a significant role of miRNAs in vascular biology and disease [87]. To date, an array of miRNAs have been also shown to be associated with AC (Table 1). Recent study demonstrated miR-125b targets transcription factor SP7 [88] or Ets1 [89] in regulating the osteogenic transdifferentiation of VSMCs; moreover, miR-125b was found to be inhibited during atherosclerotic plaque formation and was downregulated in calcified vessels [88], providing the first report concentrating on the effect of miRNAs on arterial calcification. Du et al. revealed a novel regulatory role of the miR-29/ADAMTS-7/COMP axis during arterial calcification* in vitro* and* in vivo* [90]. MiR-29a/b level was repressed in high-phosphate-induced calcifying VSMCs or blood vessels with chronic kidney disease. Additionally, Balderman et al. disclosed that the downregulation of miR-30b and miR-30c by BMP-2 increased Runx2 expression and facilitated VSMCs calcification [91]. Our group showed that miR-133a and miR-204 modulated VSMCs calcification by targeting Runx2 [92, 93]. We found that miR-133a was also significantly decreased during osteogenic differentiation of VSMCs treated with *β*-glycerophosphate. Overexpression of miR-133a inhibited VSMCs transdifferentiation into osteoblast-like cells, as proven by a decrease in ALP activity, OC secretion, Runx2 expression, and mineralised nodule formation. Conversely, the knockdown of miR-133a with a miR-133a inhibitor promoted osteogenic differentiation of VSMCs by increasing ALP activity, OC secretion, and Runx2 expression [92]. Moreover, we demonstrated that* in vitro* miR-204 was decreased in mouse aortic VSMCs during *β*-glycerophosphate-induced calcification, whereas Runx2 protein levels were elevated. Overexpression of miR-204 by transfection of miR-204 mimics suppressed Runx2 protein levels and attenuated *β*-glycerophosphate-induced osteoblastic differentiation of VSMCs, whereas miR-204 inhibition by transfection of miR-204 inhibitors significantly increased Runx2 protein levels and promoted osteoblastic differentiation of VSMCs, suggesting the role of miR-204 as an endogenous attenuator of Runx2 in VSMCs calcification.* In vivo* overexpression of miR-204 by injection of miR-204 agomirs in mice alleviated vitamin D3-induced medial artery calcification [93]. Contrary to the negative role of the above miRNAs, it has been reported that some miRNAs promote AC. Gui et al. identified that the increased expression of miR-135a^*^, miR-762, miR-714, and miR-712^*^ in VSMCs may be associated with VSMCs calcification by disrupting the potential Ca^2+^ efflux target proteins NCX1, PMCA1, and NCKX4 [94]. Mackenzie et al. proved that miR-221 and miR-222 act concomitantly to influence the trans-differentiation of murine VSMCs and contribute to the pathological process of arterial calcification* in vitro*, which may probably occur via the calcification regulators Enpp1 and Pit-1 [95]. Rangrez et al. also found that overexpressing miR-223 in VSMCs targeted Mef2c and RhoB and tended to increase VSMCs migration and calcification [96]. Taken together, all of these studies revealed the vital roles of miRNAs in osteogenic transdifferentiation and calcification of VSMCs (Table 1).

It is reported that epigenetic marks vary during aging, including a global decrease in the sufficiency of 5-methylcytosines and some histone modifications [97]. Since AC is an age-related disease, it could be speculated that those age-related changes in epigenetic marks are involved in the pathophysiology of the calcification. However, this remains to be demonstrated.

## 3. Conclusions and Perspectives

As reviewed above, epigenetic modifications such as DNA methylation, histone modifications, and miRNAs offer a new perspective in the control of gene expression, with significant applications to AC. Apart from deepening our understanding of disease mechanisms, epigenetics may be targeted for future therapies and genetic interventions. For instance, the demethylating agents 5-aza-20-deoxycytidine and procaine have been utilized in several experimental studies [98, 99]. Some HATs and HDACs inhibitors are applied in the treatment of cancer, cardiovascular disease, and neurological disorders [100, 101]. Nevertheless, their potential application is limited attributed to their nonspecific activation of genes and other genomic elements not only in diseased cells but also in normal cells. Thus, agents that are capable of regulating the epigenetic control of genes specifically in a given pathway would be much more useful. In accordance with this, therapies based on miRNAs or small interfering RNAs might be more specific and promising [102, 103]. However, studies of drugs focused on epigenetics in AC that act at the risk-factor level are still scarce. Additional studies are undoubtedly required to further elucidate how epigenetic phenomena impact the development of AC exactly and for the design of alternative treatment strategies, aimed at interfering in these epigenetic processes for the management of a variety of cardiovascular diseases related to AC.

## Figures and Tables

**Table 1:** miRNAs involved in the regulation of arterial calcification.

miRNAs	Observation	Ref.
miR-125b	Target SP7, inhibiting osteogenic transdifferentiation of VSMCs	[88, 89]

miR-133a	Target Runx2, inhibiting the osteogenic differentiation of VSMCs	[92]

miR-135a^*^/762/714/712^*^	Targets NCX1, PMCA1, and NCKX4, promoting VSMCs calcification	[94]

miR-204	Target Runx2, inhibiting VSMCs calcification	[93]

miR-221/222	Targets Enpp1 and Pit-1, contributing to arterial calcification	[95]

miR-223	Targets Mef2c and RhoB, inducing VSMCs migration and calcification	[96]

miR-29a/b	Target ADAMTS-7, inhibiting VSMCs calcification	[90]

miR-30b/30c	Target Runx2, inhibiting VSMCs calcification	[91]
